# A new role for RU486 (mifepristone): it protects sperm from premature capacitation during cryopreservation in buffalo

**DOI:** 10.1038/s41598-019-43038-4

**Published:** 2019-04-30

**Authors:** Jasmer Dalal, Pradeep Kumar, R. K. Chandolia, Shikha Pawaria, Rasika Rajendran, Suman Sheoran, Jerome Andonissamy, Dharmendra Kumar

**Affiliations:** 1 Animal Physiology and Reproduction Division, ICAR- Central Institute for Research on Buffaloes, Hisar, 125001 Haryana India; 2 Department of Veterinary Gynaecology and Obstetrics, Lala Lajpat Rai University of Veterinary and Animal Sciences, Hisar, 125001 Haryana India

**Keywords:** Steroid hormones, Infertility

## Abstract

The objective of this study was to determine the mechanism by which RU 486 (mifepristone) protects sperm to undergo premature capacitation during cryopreservation. For this, semen ejaculate (n = 20) was divided into four equal fractions and diluted using egg yolk-based extender supplemented with different concentrations of RU 486 (0, 5, 10 and 20 µM) and cryopreserved. We found that RU 486 did not impair the post-thaw sperm kinetics and motility but prevented cholesterol efflux, calcium influx, and protected CatSper channels during cryopreservation. The RU 486 protected sperm from premature capacitation which was confirmed by intracellular calcium level, expression of tyrosine phosphorylated proteins (75 and 80 kDa) and CTC (chlortetracycline) assay. Furthermore, antioxidant ability of RU 486 was reflected by the ferric reducing ability, lower production of sperm malondialdehyde and intracellular reactive oxygen species. Also, we demonstrated that RU 486 treated sperm underwent normal capacitation, zona pellucida binding and zygote cleavage indicating normal fertilizing ability of sperm. In conclusion, we report a new role of RU 486 in protecting buffalo sperm from premature capacitation during cryopreservation.

## Introduction

In spite of advancements in sperm cryopreservation, achieving optimum conception rate with frozen buffalo semen remains a challenge. Cryopreservation, being a damaging phenomenon causes the loss of viability to around 50% during this process^1,2^ and remaining motile sperm undergo premature capacitation (cry-capacitation)^3^. A variety of protocols, cryoprotectants and additives have been tried to protect sperm during cryopreservation with varied degree of success, but unable to prevent premature capacitation^4–7^. The mechanism of cryo-capacitation is not fully understood and is complicated as the onset of normal capacitation has yet to be elucidated. Therefore, capacitation-like changes in spermatozoa during cryopreservation process has fascinated and frustrated the task for reproductive biologists. Furthermore, the loss of cholesterol during cryopreservation increases the permeability of sperm membrane by stimulating several factors that allow frozen-thawed spermatozoa to undergo precocious capacitation^8^. In 2011, Strünker *et al*.^9^ and Lishko *et al*.^10^ reported that progesterone (7.7 nM) could activate a sperm-specific calcium ion channel (CatSper) resulting calcium influx which stimulate a cascade reaction responsible for capacitation. Therefore, higher progesterone concentration in egg yolk-based extender might play role in triggering signaling pathway leading to capacitation-like changes in already destabilized sperm membrane during cryopreservation. To our knowledge, no study has been attempted the use of antagonist of progesterone receptor to prevent cryo-capacitation in sperm. We report a new role of RU 486 to prevent premature capacitation of sperm during cryopreservation.

## Results

### Progesterone concentration in seminal plasma, egg yolk and semen extender

Progesterone activates CatSper channel resulting massive calcium influx that triggers cascade reaction responsible for capacitation^9,10^. Therefore, we estimated progesterone concentration in seminal plasma, hen egg yolk and semen extender. In this study, the average progesterone concentration in buffalo bull seminal plasma and hen egg yolk was 0.73 ± 0.09 ng/mL (n = 6), and 1753.77 ± 202.87 ng/mL (n = 8), respectively. The egg yolk contributed 20% of total volume of the semen extender; hence available concentration of progesterone in the extender was 438.44 ± 50.72 ng/mL. Thus, the contribution of progesterone from seminal plasma to the extender is negligible in comparison to egg yolk (Supplementary Fig. 1).

### Sperm kinetics and motility parameters

In an attempt to make the assessment of semen quality more objective, computer-assisted sperm analysis (CASA) was used and found that RU 486 did not impair (P > 0.05) the sperm kinetics and sperm motility parameters (Table 1).

**Table 1:** Effect of fortification of different concentration of RU 486 on post-thaw sperm kinetic parameters. RU 486 did not impair either sperm velocity or motility.

RU 486	VAP (µm/s)	VSL (µm/s)	VCL (µm/s)	ALH (µm)	BCF (Hz)	STR (%)	LIN (%)	TM (%)	PM (%)	RM (%)
0 µM	92.78 ± 2.23	69.63 ± 1.29	167.44 ± 5.66	7.07 ± 0.25	28.02 ± 0.89	76.59 ± 1.46	44.89 ± 1.43	43.93 ± 2.24	31.30 ± 2.28	37.75 ± 2.05
5 µM	91.70 ± 1.64	70.02 ± 1.38	162.22 ± 4.03	6.83 ± 0.24	27.42 ± 0.84	77.67 ± 1.12	46.42 ± 1.31	43.40 ± 4.69	31.55 ± 3.55	37.72 ± 4.19
10 µM	92.69 ± 2.09	70.18 ± 1.24	164.41 ± 4.97	7.15 ± 0.23	25.94 ± 0.87	76.74 ± 1.07	45.48 ± 1.23	48.81 ± 4.31	35.29 ± 3.28	42.76 ± 3.97
20 µM	92.63 ± 1.55	70.09 ± 1.27	166.24 ± 3.19	7.16 ± 0.20	27.63 ± 0.64	76.70 ± 0.71	45.02 ± 0.63	43.83 ± 3.23	31.03 ± 2.27	37.71 ± 2.91

VAP: average path velocity; VSL: straight linear velocity; VCL: curvilinear velocity; ALH: average lateral head displacement; BCF: beat cross frequency; STR: straightness; LIN: linearity; TM: total sperm motility; PM: progressive sperm motility; RM: rapid sperm motility. Values (means ± SE) in a column did not differ significantly (P > 0.05), n = 20.

### Thermal resistance test

**Figure 1:**
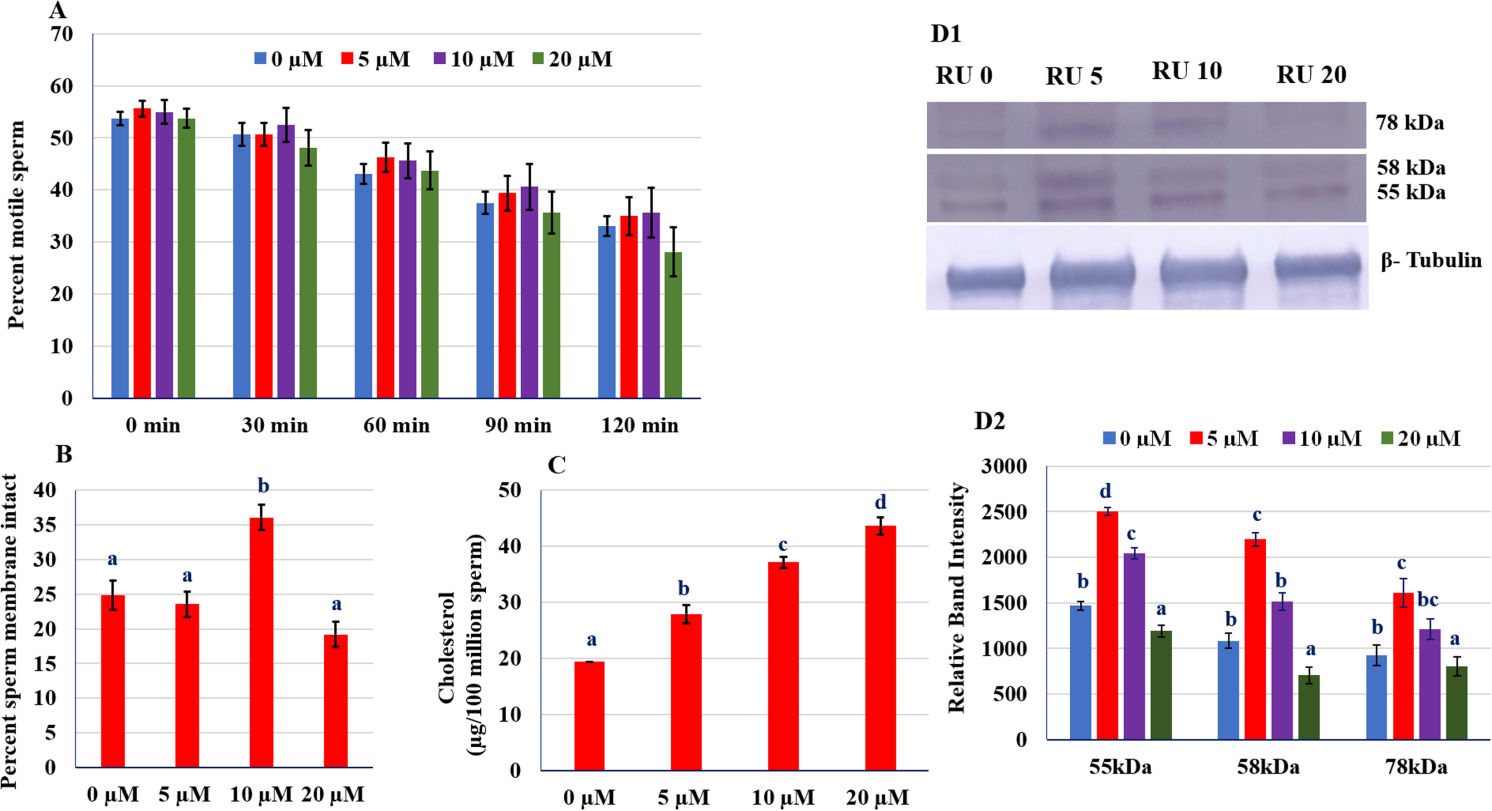
(**A**) Incubation test of RU 486 treated groups. (**B**) Plasma membrane integrity of RU 486 treated groups. (**C**) Concentration of cholesterol in post-thaw sperm. (**D1**) Western blot of CatSper 2 proteins. (**D2**) The optical intensity of CatSper proteins normalized to β-tubulin. Values with different letters (a–d) differ significantly (P < 0.05), n = 20.

The capability of sperm to survive at 38 °C was evaluated at 30 min interval for 2 h to observe any negative impact of RU 486 on post-thaw sperm motility parameters and found that there was no significance difference between the groups (Fig. 1A).

### Functional integrity of plasma membrane

Plasma membrane integrity is of key importance during freeze–thaw process and semen extender has to protect it against cryo-damage^11^. The functional intact plasma membrane was estimated by hypo-osmotic swelling test (HOST). The HOST predicts membrane integrity by determining the ability of the sperm membrane to maintain equilibrium between the sperm and its environment. The percent sperm with functional intact plasma membrane were higher (P < 0.05) in 10 µM group as compared to control and other treated groups (Fig. 1B).

### Cholesterol concentration in sperm membrane

Cholesterol, an essential component of sperm membrane, stabilizes the membrane fluidity. It is well established that during cryopreservation cholesterol efflux occurs from the sperm plasma membrane. This result into calcium influx which triggers intracellular signaling cascade associated with capacitation^8^. Therefore, we estimated sperm cholesterol and found that cholesterol efflux decreases in dose dependent manner i.e. highest cholesterol was estimated in 20 µM treated sperm and lowest in untreated sperm (Fig. 1C).

### Expression of CatSper proteins

Progesterone has been shown to trigger calcium influx into the sperm *via* activation of the sperm-specific calcium channel CatSper^9,10^. The cryopreservation decreased the quantity of the sperm membrane CatSper channel proteins^12^. To reveal the effect of RU 486 on CatSper channel expression during cryopreservation, we estimated expression of CatSper by immunoblotting. We observed three major bands of 55, 58 and 82 kDa (Fig. 1D1) and the bands’ optical densities were measured (Fig. 1D2). We found that the quantities of the CatSper proteins decreased in untreated sperm as compared to treated sperm (P < 0.05).

### Intracellular calcium status

**Figure 2:**
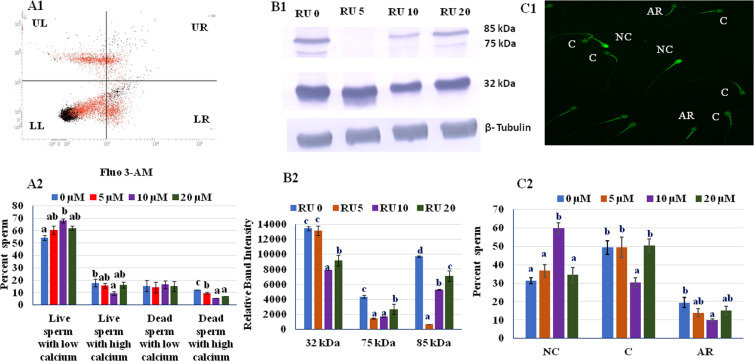
(**A1**) Flow cytometry dot plots of Fluo-3 AM/PI loaded sperm. Window lower left (LL): live sperm with low intracellular calcium, window lower right (LR): live sperm with high intracellular calcium, window upper left (UL): dead sperm with low intracellular calcium and window upper right (UR): dead sperm with high intracellular calcium. (**A2**) Intracellular calcium of sperm measured by Fluo-3 AM/PI stains. (**B1**) Western blot of tyrosine phosphorylated sperm proteins. (**B2**) The optical intensity of tyrosine phosphorylated sperm proteins normalized to β-tubulin. Values with different letters (a–c) differ significantly (P < 0.05), n = 4. (**C1**).Micrograph of sperm showing three patterns of chlortetracycline fluorescent staining (CTC): NC, non-capacitated sperm; C, capacitated sperm; AR, Acrosome reacted sperm. (**C2**) Percent mean of non-capacitated (NC), capacitated (**C**) and acrosome reacted (AR) RU 486 treated sperm. Values with different letters (a,b) differ significantly (P < 0.05), n = 20.

Physiologically, Ca^2+^ triggers multiple physiological events in spermatozoa, such as hyperactivation, capacitation, and acrosomal reaction which are essential for successful fertilization^13^ but if these phenomena occur during cryopreservation before reaching the female reproductive tract resulting poor fertilizing ability of sperm. Therefore, we estimated intracellular calcium in post-thaw sperm using fluorescent dye Fluo-3 AM by flow cytometry and found that 10 µM RU 486 treated sperm had more percent of live sperm with low calcium (Fig. 2A1,A2).

### Cryo-capacitation status of sperm

We evaluated different concentration of RU 486 for inhibition of capacitation-like changes during cryopreservation by immunoblotting of tyrosine phosphorylated proteins and chlortetracycline (CTC) assay. In the cryopreserved sperm, expression of tyrosine phosphorylated proteins, a hallmark event of capacitation, were estimated by immunoblotting (Fig. 2B1). The quantitative digital image analysis of 85 and 75 kDa bands showed greater (P < 0.05) expression of the proteins in untreated group as compared to treated groups (Fig. 2B2). The results of CTC assay showed intracellular calcium related changes during the freeze-thaw process. The CTC assay not only allows discrimination between acrosome-intact cells and acrosome reacted ones, but also divides acrosome-intact cells into two categories, i.e. uncapacitated and capacitated (Fig. 2C1). The percentage of non-capacitated sperm in 10 µM group was higher (P < 0.05) than control and other treated groups (Fig. 2C2). Further, the percent of cryo-capacitated and acrosome reacted sperm were lower (P < 0.05) in 10 µM group than other groups.

### Antioxidant activity of RU 486

**Figure 3:**
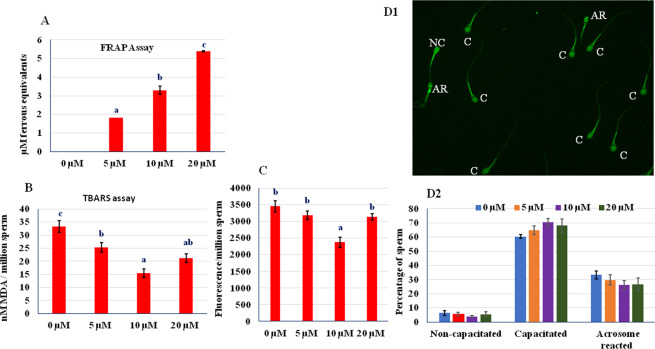
(**A**) The ferric reducing antioxidant power of RU486 fortified egg- yolk extender. Values with different letters (a–c) differ significantly (P < 0.05), n = 4. (**B**) MDA (malondialdehyde) concentration in sperm membrane. Values with different letters (a–c) differ significantly (P < 0.05), n = 20. (**C**) Intracellular reactive oxygen species (ROS) level in RU 486 treated sperm. (**D1**,**D2**.) CTC assay shows that most of the sperm irrespective of concentration of RU 486 underwent capacitation *in vitro* capacitation media.

To confirm the antioxidant activity of RU 486, ferric reducing antioxidant power (FRAP), TBARS (thiobarbituric acid reactive substances) and intracellular ROS (reactive oxygen species) assays were performed. In FRAP assay, the antioxidants act as reducing agents by donating electrons to free radicals to stabilize them. The reducing ability of RU 486 increased in a dose-dependent manner (Fig. 3A). Further, malondialdehyde (MDA), a marker of lipid peroxidation and oxidative stress, was found to be higher in untreated sperm as compared to treated sperm (Fig. 3B). Among treated group, lower (P < 0.05) concentration of MDA was found in 10 µM group. To know whether RU 486 prevents ROS production during cryopreservation we estimated intracellular ROS level and found that 10 µM group had lower level of intracellular ROS (Fig. 3C).

### *In vitro* capacitation and zona binding

**Figure 4:**
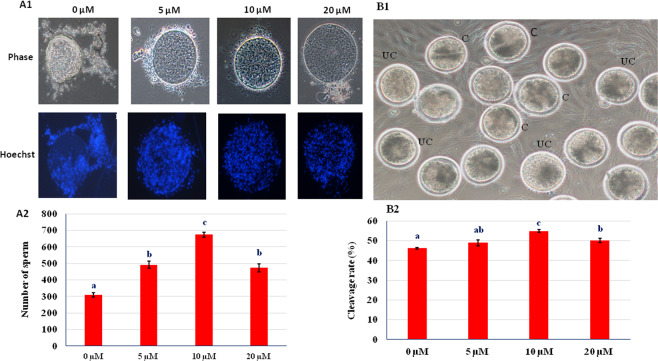
(**A1**) Micrographs showing ability of RU 486 treated sperm to attach zona pellucida in phase contrast (Magnification 10X) and fluorescence (blue) microscopes. (**A2**) Bar graph of number of sperm tightly attach to zona under different treatment. Values with different letters (a–c) differ significantly (P < 0.05), n = 5. (**B1**) *In vitro* fertilized buffalo oocytes (Magnification 10X) fertilized with RU 486 treated sperm. UC- uncleaved; C- cleaved. (**B2**) Percent cleaved zygote produced using RU 486 treated sperm.

To confirm that RU 486 treated sperm undergo normal capacitation, acrosome reaction and ability to attach with zona pellucida of oocyte; *in vitro* capacitation and zona binding assays were performed. *In vitro* capacitation test showed that all treated and untreated sperm underwent capacitation indicating that treatment does not inhibit normal sperm capacitation (Fig. 3D1,D2). The zona binding assay is based on ability of sperm to bind zona pellucida to predict the fertilizing potential of sperm^14^. The untreated sperm were found to be loosely attached to zona as compared to treated sperm (Fig. 4A1). The RU 486 treated sperm had higher (P < 0.05) zona binding ability than that of control. Among the treated groups, 10 µM groups showed higher zona binding ability than others (Fig. 4A2).

### *In vitro* fertility

The fertilizing ability of RU 486 treated sperm was evaluated by oocytes cleavage percentage after *in vitro* fertilization and we found that 10 µM group had higher (P < 0.05) cleavage rate than that of control group (Fig. 4B1,B2).

## Discussion

We estimated very low progesterone concentration (0.73 ng/mL) in buffalo seminal plasma indicating the low progesterone requirement for normal physiology of a spermatozoon after ejaculation. Surprisingly, we observed very high progesterone concentration (1753 ng/mL) in hen egg yolk used for semen extender. As 20% egg yolk is used in semen extender, the final progesterone concentration (438.44 ng/mL) in extender was as high as the physiological concentration of progesterone in oviduct^15^. In addition to progesterone, testosterone and estradiol are present in egg yolk^16^. The non-genomic progesterone receptors on sperm membrane are also activated by testosterone and estradiol but lesser intensity and induce calcium influx into spermatozoa^17^. Therefore, we hypothesized that the higher concentration of progesterone in egg yolk extender might be responsible for premature capacitation during the cryopreservation. Since higher incidence (51.3 to 69%) of sperm cryo-capacitation of using egg yolk base extender have been reported of buffalo sperm in different studies^18–20^. Hence, we anticipated anti-progesterone (RU 486) supplementation to egg yolk based extender would reduce the incidence of premature capacitation during cryopreservation process.

Our results showed that RU 486 fortification in extender did not affect the sperm kinetics and motility parameters as assessed by CASA. Our results were in line with previous observations that RU 486 has either small or negligible effect on sperm motility^21–23^. This substantiated the use of RU 486 in semen extender without hampering the post-thaw sperm motility. It is established that the cryopreservation process restructures the membranes due to phospholipids redistribution and cholesterol removal^24,25^. The loss of cholesterol from the sperm membrane results in decrease in cholesterol/phospholipids ratio which is responsible for premature capacitation during cryopreservation^20^. For the first time, the present study demonstrated that RU 486 prevents cholesterol efflux in dose dependent manner, but exact mechanism is not known. We found higher cholesterol concentration after cryopreservation in RU 486 treated sperm which indicates the cryoprotection tendered by RU 486. Since cholesterol maintains the fluidity of sperm membrane, it protects the sperm membrane proteins from denaturation^11^.

The progesterone actually activates CatSper channel resulting Ca^2+^ influx was recently reported^9,10^. During cryopreservation, there is reduction in the quantity of CatSper proteins due to protein denaturation^11^. In this study, we found expression of three variants of CatSper-2 proteins were higher in treated sperm than control. These results indicate that CatSper channels are more intact and functional in treated sperm as compared to untreated sperm confirmed by the intracellular calcium level. The intracellular calcium is key regulator of cascade reaction of capacitation, but in frozen-thaw sperm, it is negatively correlated with bull fertility^26^. In the untreated sperm, relatively higher intracellular calcium was found as compared to RU 486 treated sperm. We hypothesised that more intact and functional CatSper channels in RU 486 treated sperm after cryopreservation might be due to its antioxidant activity with lesser cholesterol efflux and sperm membrane lipid peroxidation. On the other hand, in untreated sperm more number of partially damaged CatSper channels and presence of progesterone in extender activate the channels resulting uncontrolled calcium influx and premature capacitation after cryopreservation of sperm. The ultimate biomarker of capacitation is phosphorylation of tyrosine protein in sperm^27^. This study demonstrated a decrease in phosphorylation of tyrosine proteins indicating the signal transduction pathways that lead to protein tyrosine phosphorylation is not triggered in sperm in the presence of RU 486 during cryopreservation. Earlier study has shown that sperm cryo-capacitation has been correlated with an increase in protein tyrosine phosphorylation in buffalo^18^. Finally, we tested capacitation status in treated and untreated sperm to know percent of sperm underwent premature capacitation and acrosome reaction by CTC assay and found that 10 µM RU 486 effectively prevented cryo-capacitation consistent with earlier findings^21,23^.

During sperm cryopreservation, excess free radicals are generated and progesterone promotes the generation of superoxide anion in sperm^28^. Higher production of free radicals is involved in lipid peroxidation of plasma membrane^29^. The endogenous antioxidant capacity of semen is insufficient in preventing lipid peroxidation during the cryopreservation process^30^. But, in the present study, RU 486 effectively protects cholesterol efflux, decreased intracellular free radicals and MDA from sperm membrane. This hinted us to test the reducing ability of RU 486 by FRAP assay. We, for the first time, found that ferric reducing ability of RU 486 increases in a dose-dependent manner that proving antioxidant activity of RU 486, in addition to anti-hormonal action. RU 486 consists of a progesterone-like steroid ring with a non-steroid moiety named di-methylaminophenyl (Supplementary Fig. 2) and the antioxidant activity of RU 486 resides in the non-steroid moiety^31^. Earlier, an antioxidant containing di-methylaminophenyl group named as B16 [5-(4-dimethylamino-phenyl)-2-phenyl-penta-2,4-dienoic acid] has been used in canine semen extender for storage semen at 4 °C for 72 h and found to inhibit superoxide anion by 92% and reduce total ROS production during storage^32^. From this study, it is evident that RU 486 protects sperm from premature capacitation by its anti-progesterone and anti-oxidant properties. Therefore, it is interesting to find whether RU 486 treated sperm in the oviduct would undergo normal capacitation, acrosome reaction and fertilize to the oocytes. To confirm this, we conducted three experiments *viz*. *in vitro* capacitation, zona binding assays and *in vitro* fertilization. We found that treated sperm capacitated normally and better fertilize the oocytes compared to untreated sperm. The reduced binding ability of the untreated sperm might be due to higher proportion of premature capacitated and acrosome reacted sperm losing the viability before attachment. Similar results were reported in bull^14^ and human^33^. In addition, our results are supported by the earlier finding^34^ wherein it was shown that RU 486 did not prevent fertilization or implantation in female reproductive tract. Thus, it can be anticipated that when the treated sperm reach to oviduct after artificial insemination, they are exposed to higher level of progesterone secreted by the cumulus cells and the corpus luteum^35^. RU 486 acts as a competitive progesterone receptor antagonist and presence of high concentrations of progesterone would replace RU486 from the receptor site^36^ resulting into the sperm would undergo normal capacitation and fertilization.

Taken together, the present results showed that RU 486 (mifepristone) in semen extender acts as a un-capacitation factor and protect sperm from premature capacitation during cryopreservation. Based on current and previous studies, it can be postulated that RU 486 in semen extender binds to the receptor site of progesterone, prevents activation of CatSper channel and calcium influx, minimizes tyrosine phosphorylation and prevents the initiation of capacitation during the cryopreservation process. In addition, RU 486 decreases cholesterol efflux, ROS production and lipid peroxidation in sperm due to its antioxidant activity during cryopreservation. In female reproductive tract, RU 486 is displaced from the receptor sites in presence of high concentration of progesterone and sequence of events is reverted.

## Conclusion

To the best of our knowledge, this is the first report describing a new role of RU 486 in sperm cryopreservation. Supplementation of RU 486 protects sperm from capacitation-like changes during cryopreservation without hampering the sperm post-thaw motility. Its ability to reduce ferric ions confirms its antioxidant activity in addition to anti-progesterone activity. The RU 486 treated sperm underwent normal capacitation, and attach to zona pellucida and fertilized the oocytes. These findings potentially open a new avenue of research against premature capacitation of sperm during cryopreservation.

## Methods

### Chemicals and reagents

All reagents were purchased from Sigma–Aldrich, Inc., St. Louis, MO, USA unless otherwise stated.

### Animal ethics

Animal experiments were conducted following the guidelines laid down by Institute Animal Ethics Committee, ICAR-CIRB, Hisar, and protocol approved by SERB, DST, Govt. of India.

### Estimation of progesterone in egg yolk and buffalo seminal plasma

To determine progesterone concentration, egg yolk was extracted to eliminate potential interfering substances and concentrate as described by Kozlowski *et al*.^37^. Briefly, 1 mL of PBS was added to 1 g of egg yolk and vortexed for 5 min. Thereafter, in the 100 µL of homogenised egg yolk, 400 µL of PBS was added and incubated at 37 °C for 1 h. After incubation, 500 µL of 100% ethanol was added and immediately vortexed for 1 min and allowed to incubate at room temperate for 10 min. After incubation, the sample was centrifuged at 13,000 rpm for 10 min. The supernatant was stored at −70 °C and used for progesterone estimation using progesterone enzyme immuno assay as per instruction of kit’s manufacturer (Xema-Medica, Moscow, Russia). Similarly, progesterone was estimated in seminal plasma. The standard curve was plotted using the standards and concentration for each sample is calculated from standard curve and expressed as ng/mL.

### Semen collection and cryopreservation

Semen of four Murrah buffalo bulls was collected twice a week using artificial vagina technique. Five ejaculates from each bull were used for the study. Each ejaculate was divided into four equal aliquots and extended using egg yolk- based extender fortified with different concentration of RU 486 (0, 5, 10 and 20 μM) and cryopreserved as described by Kumar *et al*.^38^.

### Sperm kinetics and motility

After cryopreservation, sperm kinetics and motility were assessed using CASA system as described earlier^39^. Before analysis under CASA, the semen sample was diluted with pre-warmed Tris buffer to give a sperm concentration of about 40 × 10^6^ spermatozoa/mL. One μL prepared semen sample was loaded in a pre-warmed (38 °C) 8 chamber Leja slide (depth 20 μm) and analyzed for sperm motility characteristics. For each sample, 5 optical fields around the central reticulum of the chamber were used to count spermatozoa. The following motion characteristics were recorded: total motility (TM, %), progressive motility (PM, %), rapid motility (RM %), straight linear velocity (VSL, µm/s), average path velocity (VAP, µm/s), curvilinear velocity (VCL, µm/s), average lateral head displacement (ALH, µm/s), beat cross frequency (BCF, Hz), straightness (STR, %) and linearity (LIN,%) of the spermatozoa. The CASA software settings were as follows: temperature = 38 °C, frame rate = 60 Hz, frames acquired = 30, minimum contrast = 35, minimum cell size = 5 pixels, cell size = 9 pixels, cell intensity = 110 pixels, progressive cells (VAP cut-off = 50μm/s, STR cut-off = 70%), slow cells (VAP cut-off = 30 μ/s and VSL cut-off = 15μ/s).

### Incubation test

Each semen sample was thawed in water bath at 37 °C for 30 s. The content of straw was transferred to 2 mL tubes maintained in a water bath at 37 °C. Sperm motility was assessed at 0, 30, 60, 90 and 120 min by using phase contrast microscope to rule out any detrimental effect of RU 486 on sperm motility during incubation period.

### Functional sperm membrane integrity

Functional integrity of sperm was evaluated by HOST as described by Swami *et al*.^40^ to know the ability of sperm to withstand stress during cryopreservation. The assay was performed by mixing 100 μL of frozen-thawed semen with 1 mL hypo-osmotic solution (0.735 g sodium citrate 2H_2_O and 1.351 g fructose in 100 mL distilled water). After incubation for 60 min at 37 °C, sperm tail bending/coiling was assessed by placing 15 μL of well-mixed sample on a warm slide (37 °C) under light microscopy at 400× magnification. At least 200 spermatozoa were observed per slide. Sperm with coiled tail after incubation was considered having intact plasma membrane.

### Determination of sperm intracellular calcium

The frozen-thawed sperm was washed to separate from semen extender and other somatic cells contamination using Bovi Pure™ (Nidacon Sweden). Intracellular calcium of sperm was analysed as per the method given by Harrison *et al*.^41^ with slight modification. Briefly, the washed sperm (∼10 million/ml) was loaded with fluorescent probe Fluo-3 AM (1 µM final concentration) and incubated for 15 min. Then, PI was added to a final concentration of 5 µM and incubated for 5 min and analyzed by flowcytometer (BD FACSVerse^TM^ San, Jose, USA). The total of 10,000 events/sample acquired at the rate of 500 events/sec. The Fluo-3 AM fluorescence was detected using a 527/32 nm band pass filter and the fluorescence of PI was detected using 586/42 nm band pass filter. The sperm population were separated into four groups: live sperm with low calcium, live sperm with high calcium, dead sperm with low calcium and dead sperm with high calcium.

### Determination of capacitation status

To confirm status of premature capacitation of sperm treated with RU 486 during cryopreservation of sperm, chlortetracycline (CTC) assay was performed^42,43^. For this, cysteine buffer (20 mM Tris, 130 mM NaCl, 5 mM cysteine; pH 7.8) was prepared and stored at 4 °C. The 750 µM chlortetracycline (Sigma-Aldrich # C4881) was prepared freshly in cysteine buffer on the day of staining and kept in light protected tube at 4 °C till use. The frozen-thawed semen was centrifuged at 140 g at 28 °C for 10 minutes to remove extender and seminal plasma. The obtained sperm pellet was re-suspended in 1 mL PBS and re-centrifuged. Consequently, the sperm pellet was re-suspended with 100 µL of PBS. A 50 µL of the sperm suspension was mixed with 100 µL CTC working solution kept for 20 minutes at 37 °C. After this, 5 µL of 12.5% glutaraldehyde (in 20 mM Tris) was added as fixative to the sperm suspension and vortexed. On clean grease free slide, 12 µL of the above sperm suspension and 6 µL glycerol was taken and cover slip was placed. A total of 200 sperm per slide were observed under FITC filter within 24 hours using fluorescence microscope (Nikon Eclipse Ti Tokyo, Japan). Sperm were evaluated according to 1 of 3 CTC staining patterns (Fraser *et al*., 1995): 1. Fluorescence over the entire head was considered non-capacitated sperm; 2. Fluorescence-free band in the post-acrosomal region was capacitated cells and 3. Low fluorescence over the entire head except for a thin bright fluorescent band along the equatorial segment was acrosome-reacted cells. A total of 200 sperm per slide were observed.

### Determination of tyrosine phosphorylation of sperm proteins

#### Extraction of sperm protein

Sperm cells from each sample were separated from semen extender and other somatic cells contamination using Bovi Pure™ (Nidacon Sweden). Proteins from the entire spermatozoa were extracted as described by Li *et al*.^44^. Briefly, the sperm pellet obtained above was solubilised in 100 µL of lysis buffer containing 4% CHAPS, 40 mM Tris-base, 75 mM DDT, 1 mM PMSF, 1 mM EDTA, 7 mM urea, 2 mM thiourea, 1 mM sodium orthovanadate and protease inhibitor cocktail @10 µL/mL. The mixture was incubated at room temperature for 60 min followed by centrifugation at 21130 g for 30 min at 4**°**C. After centrifugation, the supernatant was separated and stored at −20 °C for further use. Protein concentration was determined by Quick Start™ Bradford Protein Assay Kit (BioRad, Hercules, USA).

#### SDS-PAGE and Western blotting

For one dimensional gel electrophoresis, 10 µg of solubilized proteins were loaded per lane and resolved by SDS–PAGE (Mini-PROTEAN tetra cell (Bio-Rad) using stacking and resolving gels with 4% and 10% of acrylamide, respectively. Transfer of proteins from gel to PVDF membrane was done using iBlot^®^ Dry Blotting System (Invitrogen, USA). Immunoblotting was done by using WesternBreez® Chromogenic immunodetection system kit (Invitrogen USA), using anti-Phosphotyrosine antibody, clone 4G10® (Merck Life Science, Mumbai, India) as primary antibody. As an internal control, β-tubulin was detected using the anti-β-tubulin mouse antibody and the expression levels of the proteins were normalized to those of β-actin using myImageAnalysis software from Thermo Fisher Scientific.

### Determination of CatSper proteins

Sperm protein extraction, SDS-PAGE and western blotting were performed as described above. Immunoblotting was done by using WesternBreez® Chromogenic immunodetection system kit, anti-rabbit (Thermo Fisher Scientific, Rockford USA,), and CatSper-2 Polyclonal Antibody (Thermo Fisher Scientific, Rockford, USA) as primary antibody.

### TBARS assay

The extent of lipid peroxidation (Malondialdehyde, MDA) in samples was determined using TABARS assay kit (Cayman Chemical Company, Ann Arbor, USA) as described by Kumar *et al*.^30^. Briefly, frozen-thawed semen was centrifuged to remove seminal plasma and extender. The sperm pellet was re-suspended in PBS and again centrifuged. After centrifugation, the pellet was suspended in PBS and concentration of sperm was estimated. To each tube 100 µL of samples/standards, 100 µL of SDS solution and 4 mL color reagent was added. The mixture was kept in boiling water bath for 1 h. After 1 h, the samples and the standards were removed immediately and placed in ice bath for 10 min to stop the reaction. After cooling, the suspension was centrifuged for 10 min at 1600 × g at 4 °C. A 150 µL of the suspension was loaded into the colorimetric plate and absorbance was measured at 535 nm. The standard curve was prepared using the MDA standards and expressed as MDA µM/million of sperm.

### Estimation of cholesterol

To know whether RU 486 prevent cholesterol efflux from sperm plasma membrane, cholesterol was estimated by using cholesterol quantification Kit (Sigma Aldrich, St. Louis, MO, USA). Briefly, sperm was washed as described by Vijayalakshmy *et al*.^45^. The sperm pellet was mixed with 200 µL of chloroform: isoporpanol: IGEPAL CA-630 (7:11:0.1), vortexed and centrifuged at 13000 g for 10 min to remove insoluble material. The pellet was discarded and organic phase was air dried at 50 °C to remove any residue organic solvent. The dried lipid was dissolved with 200 mL of the cholesterol assay buffer and vortexed for two min. To 50 µL samples and standards, 50 µL of reaction mix was added to each corresponding wells to initiate reaction and kept for shaking for 1 hr at 37 °C. The absorbance was measured at 570 nm and cholesterol concentration was calculated from standard curve and expressed as µg/100 million sperm.

### Determination of ferric reducing ability

Ferric reducing antioxidant power (FRAP) of RU 486 was determined as per procedure described by Benzie *et al*.^46^ with slight modifications. Briefly, FRAP reagent is prepared by mixing 300 mM sodium acetate buffer (pH 3.6), 10 mM TPTZ solution in 40 mM hydrochloric acid, and 20 mM ferric chloride (III) solution at the ratio 10:1:1 (v/v/v), respectively. The methanol extract of extenders was used as sample for the assay. A 10 µL sample was added to 300 µL of FRAP reagent and incubated at room temperature for 3 min. The absorbance at 593 nm was measured immediately using the microplate reader. Aqueous solution of known FeSO_4_.7H_2_O concentration (0, 0.2, 0.4, 0.6, 0.8 and 1.0 mM) was used as standard curve. The values obtained were expressed as μM of ferrous equivalent Fe (II).

### Detection of intracellular reactive oxygen species (ROS)

The intracellular ROS level was measured using fluorometric intracellular ROS kit (Sigma Aldrich, St. Louis, MO, USA). The 100 µL sample and 100 µL master reaction mix was added in each well and incubated the cells in a 5% CO_2_, 37 °C incubator for one hour. The fluorescence signal was monitored at ex/em = 490/525 nm.

### Assessment of *in vitro* capacitation

Induction of *in vitro* capacitation of sperm was done as per the procedure described by Harrison *et al*.^39^. Single semen straw from each sample was thawed and centrifuged at 140 g at 28 °C for 10 min to remove semen plasma and extender. The obtained sperm pellet was re-suspended in 1 mL PBS (pH 7.4) and re-centrifuged. Supernatant was discarded and sperm pellet was re-suspended in 200 µL sperm TALP-H media^15^ and incubated for 5 h. After incubation, CTC assay was performed.

### Zona binding assay

To know ability of RU 486 treated sperm to bind zona pellucida was assessed as described previously^47^ with some modifications. The washed sperm was re-suspended in BO media without heparin to make final concentration of 2 million sperm per mL. Fresh ovaries were obtained from slaughter house and washed two to three times with PBS. Oocytes along with follicular fluid were aspirated from antrum follicles (diameter of 4 to 8 mm) of different ovaries and pooled together. Oocytes with cumulus were recovered under zoom stereo microscope and vortexed for 2 min to remove cumulus cells. The good quality cumulus free oocytes were washed with PBS and kept at 4 °C until they were incubated with spermatozoa. A small sperm droplet of 10 µL was placed in 35 mm multi-well Petri dish and an oocyte was added to each droplet. The sperm droplet with oocyte was covered with mineral oil and incubated at 38.5 °C (5% CO_2_ and 98% humidity) for 4 h. After incubation, the oocyte-sperm complex was rinsed five times in PBS to remove loosely attached spermatozoa and fixed with 2.5% glutaraldehyde in PBS for 10 min. The fixed oocyte-sperm complex was washed in PBS and stained with 1 µg/mL of Hoechst 33342 dye for 10 min. The sperm-oocyte complex was washed again in PBS to remove excess stain and placed on a glass slide under coverslip. The number of spermatozoa bound to zona pellucida was counted under epifluorescent microscope.

### *In vitro* fertilization

For assessment of fertilizing ability of RU 486 treated spermatozoa, *in vitro* maturation, fertilization and culture was performed in three replicates as described by Saugandhika *et al*.^48^. For oocyte maturation, compact cumulus oocyte complexes (COCs) with an unexpanded cumulus mass having ≥3 layers of cumulus cells and with homogenous evenly granular ooplasm were taken. The oocytes were washed 4–5 times with the washing medium (TCM-199 + 10% FBS + 0.81 mM sodium pyruvate + 100 U/mL penicillin, and 100 μg/mL streptomycin) and twice with the IVM medium (TCM-199 + 10% FBS + 5 µg/mL porcine FSH + 1 µg/mL estradiol-17β + 0.81 mM sodium pyruvate + 100 U/mL penicillin, and 100 μg/mL streptomycin). The washed COCs were then placed in 80 μL droplets (15–20 oocytes/droplet) of the IVM medium, covered with sterile paraffin oil, in a 35 mm Petri dish and cultured for 24 h in a CO_2_ incubator (5% CO_2_ in air, 90–95% relative humidity) at 38.5 °C. *In vitro* matured oocytes with expanded cumulus cells were subjected to *in vitro* fertilization. The frozen-thaw semen washed twice with washing BO medium containing 10 µg/mL heparin, 137.0 µg/mL sodium pyruvate and 1.942 mg/mL caffeine sodium benzoate. The pellet was re-suspended in 0.5 mL of the washing BO medium. The matured oocytes were rinsed in washing BO medium and transferred to 50 μL droplets (15–20 oocytes/droplet) of the capacitation and fertilization BO medium (washing BO medium + 10 mg/mL fatty acid-free BSA). The spermatozoa in 50 μL of the capacitation and fertilization BO medium were then added to the droplets containing the oocytes, covered with sterile mineral oil and placed in a CO_2_ incubator (5% CO_2_ in air) for 18 h at 38.5 °C. After the end of sperm-oocyte incubation, the cumulus cells were washed off the oocytes by gentle pipetting. The oocytes were then washed several times with the modified Charles Rosenkrans medium with amino acids (mCR2aa) as IVC medium. After 40 to 42 h of insemination, presumptive zygotes were evaluated under stereo zoom microscope for evidence of cleavage. The cleavage rate was determined by dividing the number of oocytes cleaved out of the total number of oocytes inseminated.

### Statistical analysis

All statistical analyses were carried out using IBM SPSS Statistics software (IBM Corporation, USA) for windows. Statistical significance was set at 0.05 probability level. Percent data were arcsine-square root transformed before statistical analysis. Data were analysed using one-way ANOVA and comparison of means were done by Duncan multiple range test. Results are expressed as mean ± standard error of mean. Progesterone concentration measured in seminal plasma and egg yolk was analysed by independent samples t-test. The bands intensity in western blot was calculated by using my Image Analysis software from Thermo Fisher Scientific and their means was compared by using Turkey *post-hoc* test.

## Supplementary information

supplementary information
