# Author Correction: A new role for RU486 (mifepristone): it protects sperm from premature capacitation during cryopreservation in buffalo

**DOI:** 10.1038/s41598-022-12465-1

**Published:** 2022-05-30

**Authors:** Jasmer Dalal, Pradeep Kumar, R. K. Chandolia, Shikha Pawaria, Rasika Rajendran, Suman Sheoran, Jerome Andonissamy, Dharmendra Kumar

**Affiliations:** 1 Animal Physiology and Reproduction Division, ICAR-Central Institute for Research on Buffaloes, Hisar, 125001 Haryana India; 2 Department of Veterinary Gynaecology and Obstetrics, Lala Lajpat Rai University of Veterinary and Animal Sciences, Hisar, 125001 Haryana India

Correction to: *Scientific Reports* 10.1038/s41598-019-43038-4, published online 30 April 2019

The original version of this Article contained errors.

The 32 kDa blot of Figure 2 (B1) was inadvertently duplicated as β-Tubulin in Figure 1 (D1). The original, un-processed blots for Figure 1 (D1) and Figure 2 (B1) β-Tubulin were also omitted from the Supplementary Information file. The original Fig. 1 and accompanying legend appear below. The original Supplementary Information file is linked to this correction notice.

**Figure 1:**
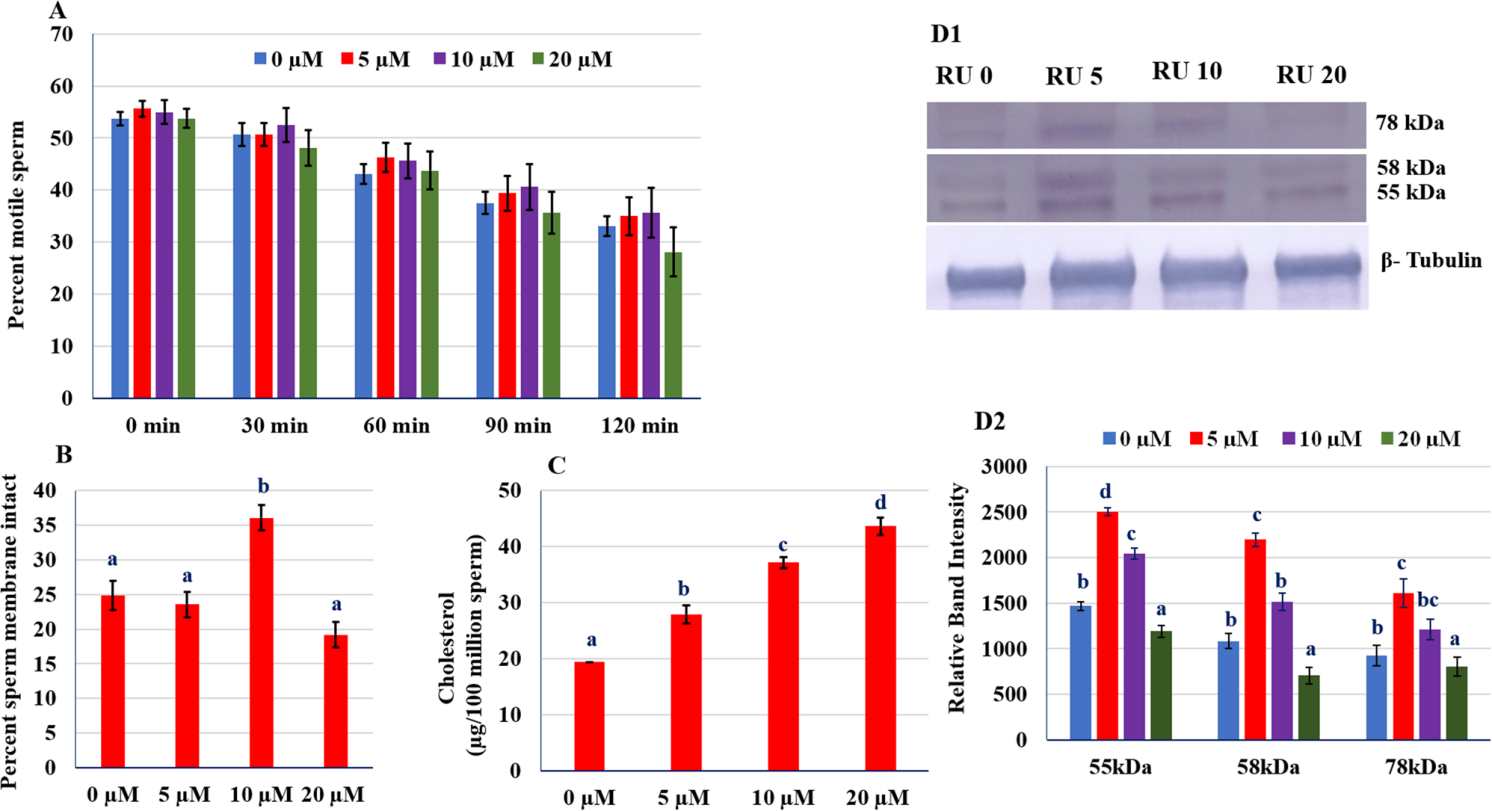
(**A**) Incubation test of RU 486 treated groups. (**B**) Plasma membrane integrity of RU 486 treated groups. (**C**) Concentration of cholesterol in post-thaw sperm. (**D1**) Western blot of CatSper 2 proteins. (**D2**) The optical intensity of CatSper proteins normalized to β-tubulin. Values with different letters (a–d) differ significantly (P < 0.05), n = 20.

The original Article has been corrected.

## Supplementary Information

Supplementary Information.

