# Supplementary material for: Author Correction: A new role for RU486 (mifepristone): it protects sperm from premature capacitation during cryopreservation in buffalo
Source: Sci Rep. 2022 May 30;12:9015. doi: 10.1038/s41598-022-12465-1 (PMC9151805; doi:10.1038/s41598-022-12465-1)
Supplement: Supplementary file 1 — Supplementary Information. [file 41598_2022_12465_MOESM1_ESM.pdf]

A new role for RU486 (mifepristone): it protects sperm from premature capacitation during cryopreservation in buffalo

Jasmer Dalal,[1,2] Pradeep Kumar,[1*] R K Chandolia,[2] Shikha Pawaria,[1] Rasika Rajendran[1] Suman Sheoran,[1] Jerome Andonissamy,[1] Dharmendra Kumar[1]

[1]Animal Physiology and Reproduction Division, ICAR- Central Institute for Research on Buffaloes, Hisar-125001, Haryana, India

[2]Department of Veterinary Gynaecology and Obstetrics, Lala Lajpat Rai University of Veterinary and Animal Sciences, Hisar - 125001, Haryana, India

***Corresponding author:**

E-mail: drpradeepkrvet@gmail.com (P Kumar)

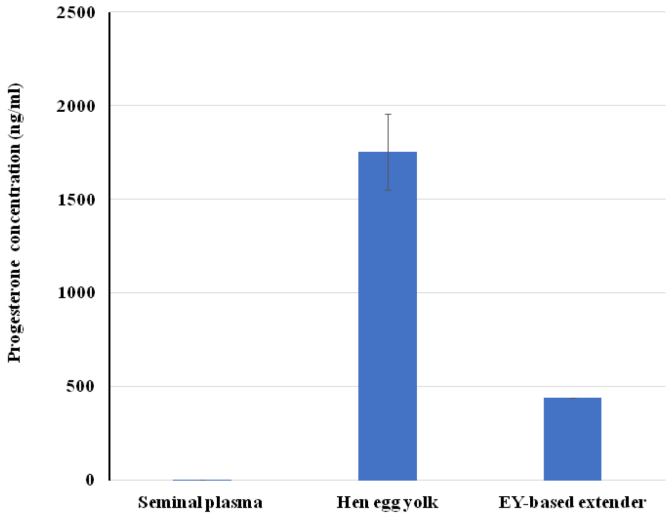

Figure S 1. Comparison of progesterone concentration in buffalo seminal plasma, hen egg yolk and egg yolk-based semen extender. Values with different letters (a–b) differ significantly ($p < 0.05$), n = 5

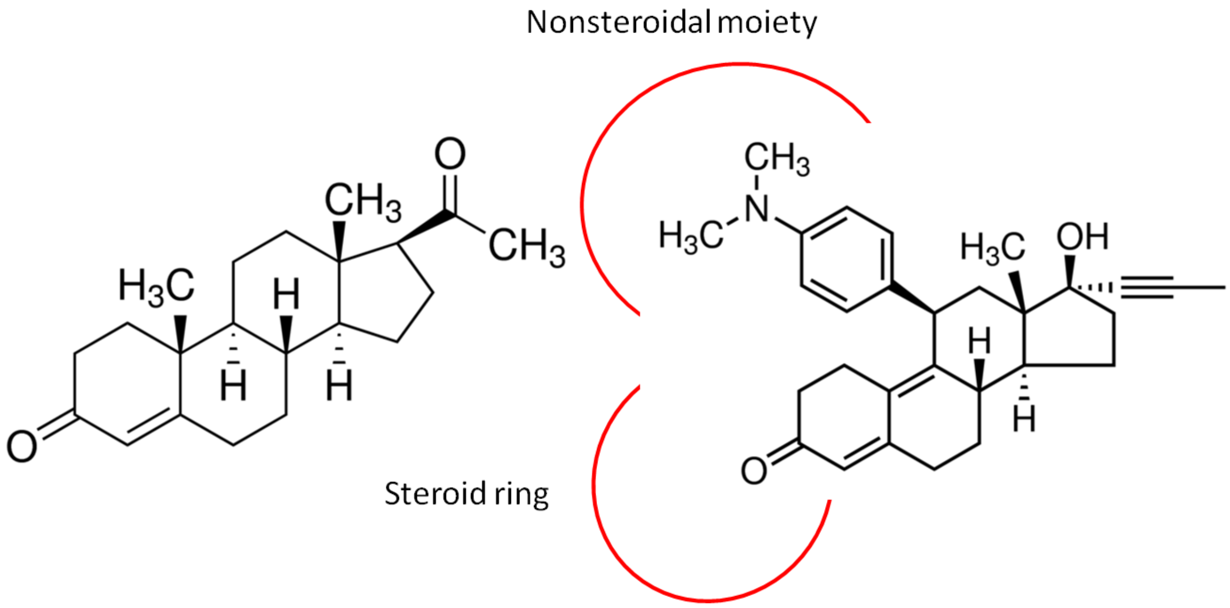

Progesterone   Mifepristone

Figure S 2. Structure of progesterone and mifepristone.

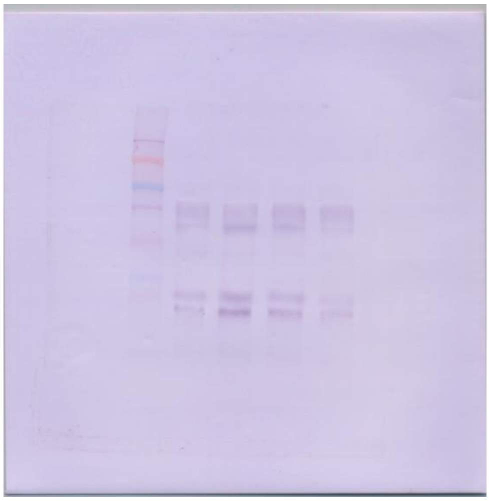

Figure S 3. Original gel for the cropped image of Fig. 1D1

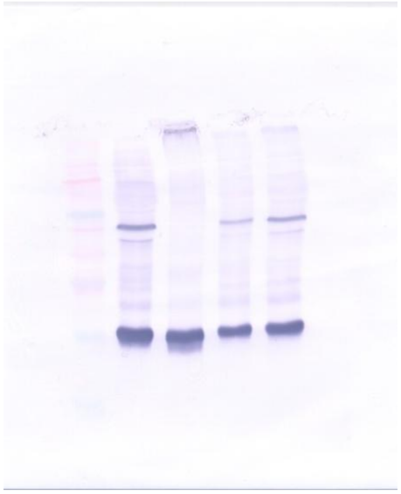

Figure S 4. Original gel for the cropped image of Fig. 2B1
